# Customized Itinerant Frequency Combs by Cascaded Nonlinear Effects

**DOI:** 10.1002/advs.77903

**Published:** 2026-09-27

**Authors:** Yi Wang, Yulong Liu, Zeji Li, Wenyan Wang, Sihan Wang, Cheng Wang, Jingwei Zhou, Jie‐Qiao Liao, Tiefu Li

**Affiliations:** ^1^ Beijing Key Laboratory of Fault‐Tolerant Quantum Computing Beijing Academy of Quantum Information Sciences Beijing China; ^2^ Key Laboratory of Low‐Dimensional Quantum Structures and Quantum Control of Ministry of Education, Key Laboratory for Matter Microstructure and Function of Hunan Province, Department of Physics and Synergetic Innovation Center for Quantum Effects and Applications Hunan Normal University Changsha China; ^3^ School of Integrated Circuits and Frontier Science Center for Quantum Information Tsinghua University Beijing China; ^4^ Hefei National Research Center for Physics Sciences at Microscale University of Science and Technology of China Hefei China; ^5^ CAS Key Laboratory of Microscale Magnetic Resonance and School of Physical Sciences University of Science and Technology of China Hefei China; ^6^ Department of Applied Physics Aalto University Aalto Finland; ^7^ Hunan Research Center of the Basic Discipline for Quantum Effects and Quantum Technologies Hunan Normal University Changsha China; ^8^ Institute of Interdisciplinary Studies Hunan Normal University Changsha China

**Keywords:** cascaded nonlinear system, itinerant frequency combs, superconducting cavity electromechanics

## Abstract

Frequency combs are pivotal in nonlinear optics, with extensive applications in modern optics and precision measurement. Precise control over their critical parameters is essential to enhance performance and expand the range of applications. In this work, we develop a cascaded third‐order nonlinear system to create an on‐demand and customized itinerant frequency comb (IFC). Unlike traditional microcavity Kerr combs, the IFC decouples the comb center frequency from the cavity resonance, thereby enabling arbitrary spectral width scaling and flexible comb spacing adjustments. In particular, we demonstrate the real‐time engineering of the central frequency of IFCs, thereby enabling the splicing and subdivision of frequency combs. These advances have the potential to enhance the performance of existing applications and expand new application scenarios by harnessing cascaded nonlinear effects.

## Graphical abstract

A cascaded third‐order nonlinear system generates an itinerant frequency comb with the comb center frequency decoupled from the cavity resonance. This enables arbitrary spectral scaling, flexible spacing adjustments, and real‐time central‐frequency engineering, allowing comb splicing and subdivision. The resulting versatility and reconfigurability open new paradigms for nonlinear optics and precision metrology.

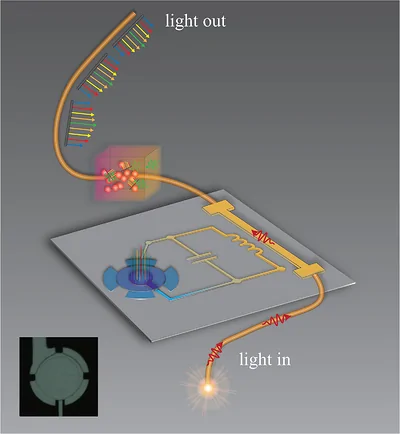

## 1 Introduction

Frequency combs [1, 2, 3, 4, 5] are optical spectra consisting of a series of frequency components with identical spacing, and their generation primarily relies on various nonlinear physical effects. For example, microcavity optical frequency combs [6, 7, 8, 9, 10] can be generated by the continuous laser pumping of a high‐quality microcavity [11, 12]. The underlying principle is to use the nonlinear effect of four‐wave mixing [13, 14, 15], and they have the characteristics of miniaturization, integration, and low power consumption [16, 17, 18, 19]. Recently, optomechanical frequency combs (OMFC) have been generated in cavity optomechanical systems [20, 21, 22, 23, 24] based on the effective Kerr‐type nonlinear effect [25, 26, 27]. The OMFC not only has the above‐mentioned features, but also has the potential to be compatible with the widely used microwave‐frequency‐band communication fields [28, 29, 30].

Currently, various crucial applications of the frequency combs in accurate time and frequency standards [31] have been proposed. These applications include atomic clock [32, 33, 34, 35, 36], optical communication [37, 38, 39], molecular fingerprinting [40, 41], and optical metrology [42, 43]. In atomic clock, frequency combs are used to excite atomic transitions and calibrate the transition frequencies [35]. Both processes require that the frequency combs match precisely the atomic transition frequency [32, 33, 34]. Frequency combs can be used to identify and differentiate various substances via fine spectral structures, such as molecular fingerprinting [40, 41]. Furthermore, the applications of frequency combs in optical metrology require a large number of spectral lines [42, 43]. Therefore, how to precisely control the key parameters, such as the central frequency, comb tooth spacing, and the number of spectral lines of the frequency comb, becomes a very important and highly desired task in this field.

Cascade systems [44, 45, 46] can be used to precisely control complex physical processes and improve overall system performance by modulating subunits and their synergistic effects [47, 48, 49, 50]. For example, cascaded synchronization [51] can be achieved in complex networks based on the synergistic effects of subsystems [52]. The performance of quantum cascade lasers [53, 54] can be significantly enhanced by optimizing the cascade structure of the active region [55, 56, 57]. In the field of many‐body physics, it is advantageous to achieve precise control over collective dynamics by tuning subunits and their synergistic effects [58, 59, 60, 61, 62]. Additionally, hybrid quantum networks that incorporate cascaded and interconnected heterogeneous architectures can harness the advantages of sub‐quantum nodes, presenting a promising pathway for universal quantum information processing, such as distributed quantum computing [63, 64, 65, 66, 67].

In this work, we constructed a cascaded nonlinear system consisting of a cavity electromechanical device and a high‐electron‐mobility transistor (HEMT). This system allows for the modulation of both individual and combined nonlinear effects. We demonstrated real‐time engineering of the central frequency of itinerant frequency combs (IFCs), which is a significant difference over traditional microcavity or optomechanical Kerr combs that have a fixed center frequency. By leveraging the cascaded third‐order nonlinear effect, we achieved real‐time adjustment of both the comb tooth positions and the number of generated comb lines. This capability enables flexible subdivision and splicing of IFCs. In particular, all operations are conducted at single‐photon probe powers. Precise control of critical parameters in frequency combs can significantly enhance their performance and broaden their range of applications.

## 2 Results

### 2.1 Cascaded Nonlinear Systems and Customized Itinerant Frequency Combs

**FIGURE 1:**
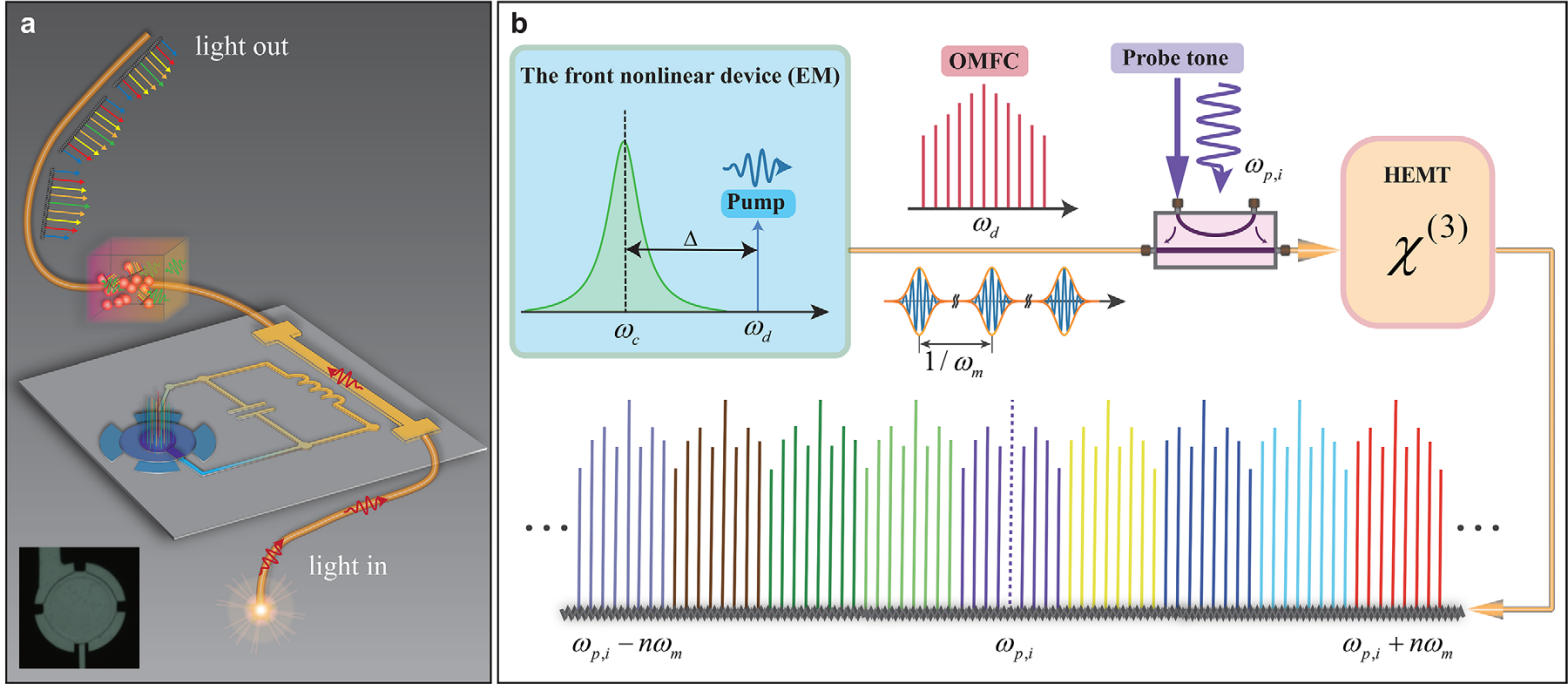
(a) Cartoon of the physical realization of the cascaded nonlinear system, which is composed of a superconducting cavity electromechanical system and a nonlinear device, e.g., a high‐electron‐mobility transistor (HEMT). The inset is an optical micrograph of the mechanical oscillator. The coherent single‐frequency microwave tone enters the electromechanical system, where it undergoes nonlinear interactions. The output from this system serves as the input for the cascaded nonlinear HEMT device, which exhibits third‐order nonlinearity. (b) Generation principle of the customized IFCs. The output of the electromechanical device, which is an OMFC with central frequency $\omega_{d}$ and tooth spacing $\omega_{m}$, combined with another weak single‐frequency coherent tone with frequency $\omega_{p}$, serves as the input for the second nonlinear device. The nonlinear interaction between the probe coherent tone and the OMFC generates new IFCs with the central frequency $\omega_{p}$ and comb tooth spacing $\omega_{m}$. Each color represents an IFC.

The constructed cascaded nonlinear system, as shown in Figure 1a, consists of a superconducting cavity electromechanical device (Figure S1 and Section S1) and a high electron mobility transistor (HEMT). The front nonlinear system is the superconducting cavity electromechanical device, which is a typical optomechanical configuration in the radio‐frequency range. It is composed of superconducting microwave circuit resonators and a drum‐type mechanical resonator fabricated from thin aluminum films. The aluminum suspended membrane and the aligned bottom electrode form a mechanical capacitor, which is highly sensitive to the vibrational displacement of the mechanical modes, particularly those with central drumhead‐type mode shapes.

The coherent pump tone (see Figure 1a) initially enters the microwave cavity of the electromechanical device. The electromechanical radiation pressure interaction acts as an effective third‐order nonlinearity. This interaction enables the pump tone to undergo a spontaneous parametric down‐conversion (SPDC) process, which in turn generates frequency combs. Subsequently, the output microwave tones serve as seed comb inputs into the second nonlinear device within the cascaded structure. In this work, the second nonlinear device is a HEMT that operates in the nonlinear regime, further amplifying and processing the seed electromechanical comb tones. This configuration introduces a cascaded third‐order nonlinear process for the transmitted seed comb, facilitating enhanced interactions and further manipulation of the frequency comb characteristics.

Specifically, the generation of customized frequency combs is illustrated in Figure 1b. When a single‐frequency microwave coherent tone with frequency $\omega_{d}$ and amplitude $S_{in}$ is introduced as input, the evolution of the microwave cavity field over time, influenced by the optomechanical nonlinearity, can be described as (see Section S4)

(1) $$a\left(t\right)=\sum_{n=-\infty }^{\infty }a_{n}e^{-i\omega_{d}t}e^{in\omega_{m}t}.$$

Here, $\omega_{m}$ is the resonance frequency of the mechanical mode. The amplitude of the down‐converted n‐th order sideband in an electromechanical system can be expressed as

(2) $$a_{n}=\sqrt{\kappa_{e}}S_{in}\sum_{k=-\infty }^{+\infty }i^{n}\frac{J_{n-k}\left(\xi \right)J_{k}\left(-\xi \right)}{\kappa /2-i(\Delta_{dc}-k\omega_{m})},$$

where $J_{k}\left(\xi \right)$ is the Bessel function of the first kind, and $\xi =2gB_{0}/\omega_{m}$ with g and $B_{0}$ being the single‐photon coupling strength and the resonance amplitude of the mechanical mode, respectively. The detuning is defined as $\Delta_{dc}=\omega_{d}-\omega_{c}$, where $\omega_{c}$ is the resonant frequency of the microwave cavity field. The coefficient $\kappa =\kappa_{i}+\kappa_{e}$ is the total cavity damping rate, with $\kappa_{i}$ and $\kappa_{e}$ being the coupling rates associated with the intrinsic and the external thermal reservoir, respectively.

In this experiment, the cavity electromechanical device is placed in a dilution refrigerator that can cool well below the superconducting transition temperature of aluminum (Figure S2 and Section S2). Based on $S_{21}$ detection (using a vector network analyzer) and power spectral density measurement (using a power spectrum analyzer), we can obtain the resonance frequency of the mechanical oscillator $\omega_{m}/2\pi \approx 9.148$ MHz, with a damping rate $\gamma_{m}/2\pi \approx 103.15$ Hz, corresponding to a mechanical quality factor of 8.86$\times 10^{4}$. The microwave mode has the resonant frequency of $\omega_{c}/2\pi \approx 4.96$ GHz and the damping rate of κ/2π=346.2 kHz, corresponding to a cavity quality factor of 1.43$\times 10^{4}$ (Figure S3). By comparing the damping rate κ of the microwave cavity and the frequency $\omega_{m}$ of the mechanical oscillator, we confirm that the electromechanical system works in the resolved sideband regime $\left(\omega_{m}>\kappa \right)$ [68, 69, 70, 71, 72]. The vacuum gap distance between the capacitor's plates determines the single‐photon coupling strength of the electromechanical interaction g. From the measurements based on a frequency modulation technique [73, 74], the single‐photon coupling strength is calibrated as g/2π≈62.49 Hz (Figure S4 and Section S3). The device parameters are summarized in Table S1 (see Section S3).

The output frequency comb with the central frequency $\omega_{d}$ and comb tooth spacing $\omega_{m}$ flies along the waveguide and then encounters another weak probe coherent tone with central frequency $\omega_{p}$. As schematically shown in Figure 1b, these two tones serve as the input field for the second nonlinear device. The total electric fields injected into the cascaded second nonlinear system are described as:

(3a) $$\begin{array}{cc} E_{out} & =\sum_{n=-\infty }^{\infty }E_{n}a_{out,n}e^{-i\left(\omega_{d}-n\omega_{m}\right)t}+c.c., \end{array}$$

(3b) $$\begin{array}{cc} E_{fly} & =E_{p}e^{-i\omega_{p}t}+c.c., \end{array}$$

where

(4a) $$\begin{array}{cc} a_{out,0} & =S_{in}-\sqrt{\kappa_{e}}a_{0}, \end{array}$$

(4b) $$\begin{array}{cc} a_{out,n} & =\sqrt{\kappa_{e}}a_{n}\;\;\left(n\neq 0\right), \end{array}$$

and $E_{n}=\sqrt{\hbar \left(\omega_{d}-n\omega_{m}\right)/\left(V\varepsilon_{0}\right)}$ with V and $\varepsilon_{0}$ being the volume of the microwave field and vacuum permittivity, respectively. $E_{p}$ represents the amplitude of the weak probe tone. These flying photons enter the second nonlinear system interfacing with the nonlinear medium of HEMT. The polarization response method can be employed to depict the relationship between the polarization intensity P and the electromagnetic field E, which is given by (see Section S5)

(5) $$\begin{array}{ccc} P_{f}^{\left(3\right)}\left(t\right) & = & 3\varepsilon_{0}\sum_{n,l}\{\Gamma_{1}^{\left(3\right)}E_{n}^{*}E_{l}E_{p}e^{-i\left[\omega_{p}+\left(n-l\right)\omega_{m}\right]t}\\ & & +\;\Gamma_{2}^{\left(3\right)}E_{n}E_{l}^{*}E_{p}e^{-i\left[\omega_{p}-\left(n-l\right)\omega_{m}\right]t}+c.c.\}, \end{array}$$

where the parameter 3 represents the intrinsic commutation symmetry of the third‐order nonlinear polarizability and

(6a) $$\begin{array}{c} \Gamma_{1}^{\left(3\right)}=\chi^{\left(3\right)}\left(-\omega_{d}+n\omega_{m},\omega_{d}-l\omega_{m},\omega_{p}\right)a_{out,n}^{*}a_{out,l}, \end{array}$$

(6b) $$\begin{array}{c} \Gamma_{2}^{\left(3\right)}=\chi^{\left(3\right)}\left(\omega_{d}-n\omega_{m},-\omega_{d}+l\omega_{m},\omega_{p}\right)a_{out,n}a_{out,l}^{*} \end{array}$$

with $\chi^{\left(3\right)}$ being the third‐order polarizability.

Equation (5) elucidates the fundamental mechanisms underlying the generation of customized frequency combs induced by cascaded nonlinearity. Specifically, according to Equation (5), the nonlinear interaction between the probe coherent tone (with frequency $\omega_{p}$) and the seed frequency comb (with central frequency $\omega_{d}$) results in the creation of a new IFC. Under the interaction of cascaded nonlinear effects, the IFC retains the lower finesse (comb tooth spacing $\omega_{m}$) of the seed frequency comb while inheriting the advantage of arbitrarily adjustable probe frequency $\omega_{p}$. Therefore, these cascaded frequency combs exhibit the remarkable ability to adjust their central frequency in real time as needed. This capability provides the physical mechanism to realize the splicing and subdivision of the IFC. Based on the splicing and subdivision techniques of the IFC, we achieve dynamic and real‐time control over three pivotal parameters: precise tuning of the central frequency, flexible adjustment of the comb teeth spacing, and seamless splicing of the spectral lines. This breakthrough has enabled the development of a customized IFC that primarily covers the entire radio‐frequency range of superconducting circuits, specifically from 4 to 8 GHz, which is the main frequency band for microwave photonics. It is worth mentioning that the system will also generate a frequency comb of the central frequency $2\omega_{d}-\omega_{p}$ under the modulation of the third‐order nonlinear polarization effect, and that the frequency comb is symmetrical with the IFC about the pump frequency $\omega_{d}$ (Figure S5 and Section S5).

### 2.2 On‐Demand Adjustable Itinerant Frequency Comb

The OMFC is a physical resource with broad application prospects in the microwave frequency band. The comb tooth spacing of the OMFC is determined by the mechanical frequency $\omega_{m}$, while the central frequency is constrained by the pump frequency $\omega_{d}$. Additionally, the number of spectral lines is limited by the optomechanical coupling strength and pump power. In this section, we present the experimental observations of the evolution of the OMFCs within the cascaded nonlinear system. Leveraging the cascaded nonlinearity structure, we further demonstrate a customized frequency comb with adjustable central frequency, tooth spacing, and number of spectral lines. This adaptability has the potential to enhance the performance of existing applications and broaden the scope of future applications.

**FIGURE 2:**
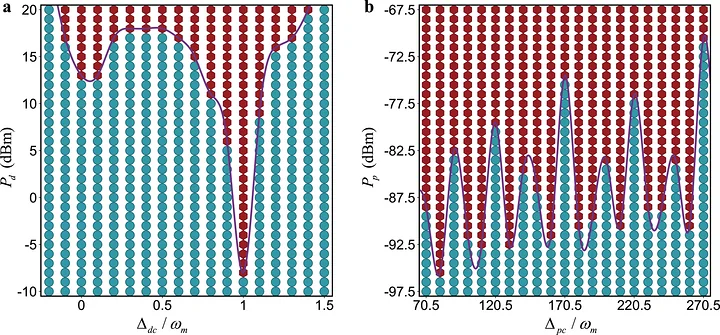
(a) Different regimes (stationary state, frequency comb) of the electromechanical system in a parameter map of the detuning $\Delta_{dc}/\omega_{m}$ and the pump power $P_{d}$. (b) Different regimes (stationary state, frequency comb) of the electromechanical system in a parameter map of the detuning $\Delta_{pc}/\omega_{m}$ and the power $P_{p}$ of the probe tone. Dark‐red hexagons indicate the frequency combs, while a dark‐green circle indicates the stationary state.

**FIGURE 3:**
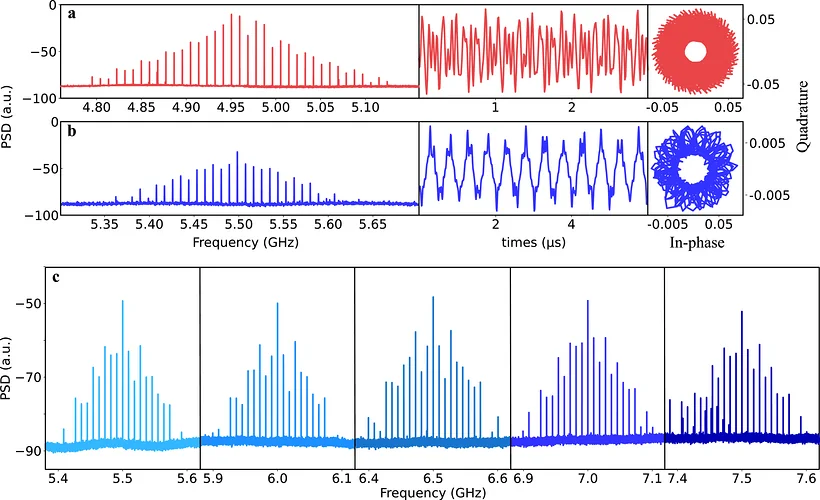
(a) The left‐hand column displays the spectra of the OMFC with a pump power of 18 dBm and the IFC with a probe power of 0 dBm. The middle column shows the time‐domain features. The right‐hand column exhibits the in‐phase and quadrature amplitudes. (b) The left‐hand column displays the spectra of the IFC with a probe power of 0 dBm. The middle column shows the corresponding behavior in the time domain. The right‐hand column exhibits the in‐phase and quadrature amplitudes. (c) The IFCs are generated through cascaded third‐order nonlinear interaction between the OMFC and the probe tones. From left to right, the central frequencies of the probe tones are sequentially 5.5, 6.0, 6.5, 7.0, and 7.5 GHz.

Experimentally, we first examined the dependence of the spectral evolution in the traditional electromechanical system on the pump power. As shown in Figure 2a, the dark‐green circle indicates that the electromechanical system stays in the steady state, and thus only a single peak located at the pump frequency $\omega_{d}$ is recorded by the spectrum analyzer. When the pump power $P_{d}$ exceeds the instability threshold, the mechanical resonator experiences a self‐excited oscillation, becoming unstable, and an OMFC is generated, which is denoted by the dark‐red hexagon. Typical spectra of OMFC are shown in Figure 3a, where the central frequency is the driving frequency $\omega_{d}$ and the comb teeth spacing is the mechanical frequency $\omega_{m}$. The time‐domain signal indicates that the OMFC has excellent coherence. Figure 2a shows that the smallest threshold pump power to enter the unstable state exists when $\Delta_{dc}/\omega_{m}=1$, which indicates that the system is most likely to enter an unstable state at the exact blue sideband. As the pump frequency $\omega_{d}$ goes away from the optimal pump working point, the required pump power dramatically increases. The purple solid line describes the boundary of the threshold power between the steady state and the OMFC generation. When operating the pump in the red sideband region, instability requires a sharp increase in pump power that often exceeds the device safety limits, potentially triggering unwanted phenomena such as the breakdown of superconductivity.

For the cascaded nonlinear system as schematically shown in Figure 1a, the OMFC will be injected into the second nonlinear system to serve as the seed signal. To investigate the impact of the cascaded nonlinear effects on OMFC, we feed the HEMT with another probe tone that is significantly detuned from the microwave resonant frequency $\omega_{c}$. The dependence of the spectral evolution on the power of the probe $P_{p}$ tone is shown in Figure 2b. The dark‐green circle indicates that only one peak is visible, while the dark‐red hexagon signifies that more than four equally spaced spectral lines are observable. Our experiment demonstrates that the probe tone operating at the single‐photon power level is sufficient to generate the frequency comb, where the detuning $\Delta_{pc}=\omega_{p}-\omega_{c}$ between the central frequency of the comb and the microwave resonant frequency exceeds the cavity linewidth κ by over three orders of magnitude ($\Delta_{pc}/\kappa >1000$). This far‐detuning confirms that the frequency combs are predominantly governed by nonlinear photon‐photon interactions rather than microwave cavity mode properties, effectively decoupling the comb spectrum from the microwave resonator. Consequently, the frequency comb can itinerate through the waveguide like a flying photon. We wish to clarify that the seesaw‐like trend in Figure 2b does not arise from a fundamental physical mechanism but stems primarily from random fluctuations. Because the probe power used in this measurement is close to the single‐photon level, the system operates near its measurement detection threshold, making the boundary sensitive to random experimental noise and background fluctuations.

In Figure 3b, we present the spectrum of the IFC under a probe power of 0dBm. Due to the high pump power, the IFC spectrum exhibits a ${\text{sech}}^{2}$‐like envelope [75]. Notably, the central frequency of the IFC aligns with the probe frequency (5.5GHz), and $\Delta_{pc}/2\pi \approx 0.54\;\text{GHz}\gg \kappa /2\pi =346.2\;\text{kHz}$. This shows IFC can be free of the fixed central frequency. This phenomenon has been observed within an ultra‐wide bandwidth range, e.g., covering 4‐8 GHz. A time‐domain signal resembling a sine wave suggests good coherence of the IFC. As shown in Figure 3c, we show the IFCs generated by multiple probe tones far from the microwave cavity under the cascaded third‐order nonlinear effects. From left to right, the frequencies of the weak coherent probe tone that generated the IFCs are sequentially 5.5, 6.0, 6.5, 7.0, and 7.5 GHz. When the power $P_{p}$ of the probe tone remains constant, shifting the probe tone frequency $\omega_{p}$ away from the microwave cavity does not eliminate the spectral signatures of the itinerant frequencies. The frequency comb dynamically propagates through the waveguide by tracking the probe tone frequency. This confirms that the cascaded architecture enables tunable synthesis of the IFCs across arbitrary microwave frequency bands. Therefore, we can achieve a customized frequency comb by adjusting the frequency $\omega_{p}$ and the power $P_{p}$ of the probe tone. Under the interactions between cascaded nonlinearity, the newly generated frequency comb exhibits two distinct characteristics: first, the central frequency of the new comb shifts to the resonant frequency of the probe light; second, the input power of the probe field can be as low as the single‐photon level.

### 2.3 Phase Coherence of Itinerant Frequency Combs

In this section, we perform a systematic characterization of IFC coherence, with core performance indicators such as comb linewidth, long‐term stability and phase noise characteristics comprehensively analyzed.

**FIGURE 4:**
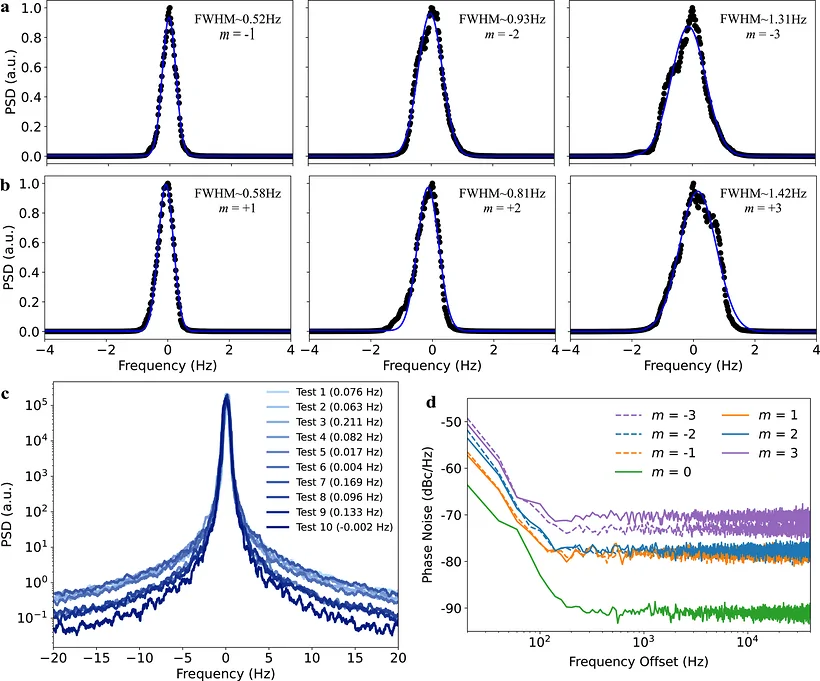
Normalized output spectrum (black dots) and the corresponding Voigt fit (blue curve) as a function of linewidth for (a) the negative‐order (m=−1,−2,−3) and (b) the positive‐order (m=+1,+2,+3) IFCs. (c) Long‐term stability of the m=+1 comb tooth. Solid lines (light to dark blue) represent 10 test runs over one hour. The legend lists the peak frequency offsets, exhibiting a standard deviation of approximately 0.067 Hz. (d) Phase noise spectra of the IFC.

Figure 4a,b shows the normalized output power spectra alongside the corresponding Voigt fitting curves for different orders (m=±1,±2,±3). Experimental observations reveal that the full width at half maximum (FWHM) of the comb lines exhibits a clear linear broadening trend with increasing absolute order |m|: for |m|=1, the FWHM is approximately 0.52-0.58Hz; as the order increases to |m|=2, the FWHM grows to 0.81-0.93Hz; and for |m|=3, the linewidth further broadens to 1.31-1.42Hz. The physical mechanism underlying this broadening behavior can be attributed to the quasi‐static slow drift (δΩ) present in the driving modulation frequency. This drift introduces an order‐dependent frequency shift in the m‐th comb line (given by $\delta \omega_{m}=m\delta \Omega$), which induces inhomogeneous broadening and causes the linewidth standard deviation and FWHM to strictly follow the theoretical relation ${\text{FWHM}}_{m}\propto \left|m\right|\sigma_{\Omega }$ [76]. Figure 4c displays the superimposed spectra of the m=1 comb line over a 1‐h testing period, comprising 10 independent measurements taken at approximately 360‐s intervals. Throughout the hour‐long observation, the comb line not only maintains a highly consistent spectral profile but also exhibits an extremely small peak frequency shift standard deviation of only 0.067Hz.

Figure 4d illustrates the measured single‐sideband phase noise power spectral density of the IFC across various comb orders (m=0,±1,±2,±3). First, the noise profiles of symmetrical orders (+m and −m, shown in solid and dashed lines) overlap almost perfectly and exhibit a stepwise elevation. These behaviors indicate that sideband phase noise is governed predominantly by the mechanical oscillator rather than pump tone fluctuations or incoherent thermal noise (Figure S6 and Section S6). Specifically, the spectral symmetry reflects a shared mechanical drive, while the stepwise elevation demonstrates that higher‐order comb lines coherently accumulate and linearly map the mechanical phase jitter ($\delta \phi_{m}=m\delta \theta$). Finally, the parallelism of the noise traces and the distinctly lower noise level of the central comb line (m=0, green curve) indicate that m=0 inherits the phase stability of the highly coherent probe light, remaining effectively decoupled from the mechanical fluctuations that govern the higher‐order sidebands.

Table 1 summarizes the key performance metrics of state‐of‐the‐art optomechanical frequency combs (OMFCs), superconducting‐circuit frequency combs (SFCs), and magnonic frequency combs (MFCs), including phase noise, tuning range of central frequency, power threshold, comb lines, and comb spacing. Compared with previously reported OMFCs, the proposed IFC exhibits a lower threshold power. Moreover, conventional frequency combs are typically restricted to a narrow blue‐detuned band, and physical constraints fundamentally prevent their scalability to arbitrary microwave frequencies. In contrast, an IFC overcomes these limitations, enabling flexible comb‐line generation across arbitrary microwave frequencies. This broad tunability facilitates refined comb manipulation, such as splicing and subdivision, by simply adjusting the probe tone frequency.

**TABLE 1:** Parameters of different frequency combs. The symbol “ —” indicates that the parameter value was not explicitly reported, while the tuning‐range and comb‐lines data were manually extracted from representative figures in the literature. Owing to space constraints, we list only a selection of references, and we acknowledge that this does not encompass all relevant work.

Type	Phase noise	Tuning range of central frequency	Power threshold	Comb lines	Comb spacing
OMFC [21]	−100 dBc/Hz	—	1.53 mW	867	50.22 MHz
OMFC [22]	—	3.6 MHz	0.251 μW	36	756 kHz ‐ 1.75 MHz
OMFC [23]	—	—	231 μW	260	77.19 ‐ 90.17 MHz
OMFC [28]	—	—	3 mW	35	118 kHz
OMFC [75]	−163.1 dBc/Hz	—	—	126	7.6 MHz
OMFC [76]	−85 dBc/Hz	54.6 MHz	10 nW	10	9.1 MHz
OMFC [77]	−120 dBc/Hz	—	190.5 mW	11	8.04 MHz
OMFC [78]	−40 dBc/Hz	—	82 μW	38	80.1 kHz
OMFC [79]	−132 dBc/Hz	—	120 μW	42	1.655 GHz
OMFC [80]	−85 dBc/Hz	—	200 μW	12	41.95 MHz
OMFC [81]	—	9.6 MHz	13.5 mW	25	8.0 MHz
SFC [82]	—	—	63 μW	46	390 MHz
SFC [83]	—	—	1.6 μW	327	59.738 MHz
SFC [84]	—	—	40 pW	54	0.75 GHz
SFC [85]	—	—	—	6	50 MHz
SFC [86]	—	39 MHz	15.85 μW	11	6.4 MHz
MFC [87]	—	—	0.2 mW	4	400 ‐ 700 MHz
MFC [88]	—	—	20.9 μW	33	0.1 MHz
MFC [89]	—	—	55 mW	21	10.08 MHz
MFC [90]	—	—	10 mW	6	0.5 ‐ 2 GHz
IFC (This work)	−80 dBc/Hz	RF range (GHz)	4.47 nW	≥500	9.148 MHz

### 2.4 Itinerant Frequency Comb Splicing and Subdivision

**FIGURE 5:**
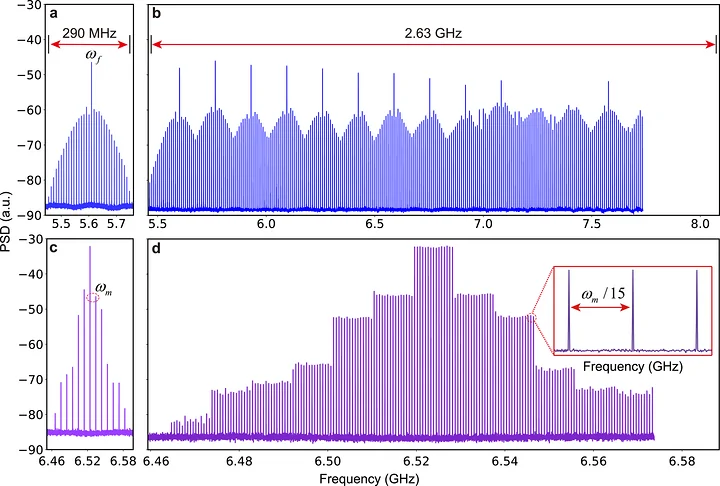
The splicing and subdivision of the IFCs. (a) A frequency comb with central frequency 5.6 GHz and range 290 MHz. (b) An ultra‐wide frequency comb obtained by splicing 15 IFCs together. (c) A frequency comb with central frequency 6.52 GHz and comb tooth spacing $\omega_{m}$. (d) A hyperfine frequency comb obtained by subdividing the tooth spacing of the IFC.

We proceed to experimentally demonstrate the splicing and subdivision of IFCs by utilizing the two characteristics previously highlighted. Concretely, the seed frequency comb is generated by driving the superconducting cavity electromechanical device with a 10 dBm pump tone at the blue sideband with a resonance of 4.968634 GHz. Due to the cascaded nonlinear interaction, the probe tone and the seed comb are mixed, resulting in the generation of new frequency components and forming the IFC with a central frequency of 5.61 GHz, as illustrated in Figure 5a. This IFC can be used as the basic unit of frequency comb splicing. Subsequently, the seed comb remained fixed and was co‐injected with 15 probe tones into the second nonlinear system. Both the powers and frequencies of these probe tones were independently tunable. By specifically calibrating the probe power, each probe tone generates the IFC that is the same as the splicing base unit of Figure 5a. Meanwhile, the frequency interval between probe tones is set to $18\omega_{m}$. Each probe tone interacts with the seed frequency comb to generate a separate IFC. The 15 IFCs (bandwidth of 290 MHz) can be seamlessly spliced into an ultra‐wide frequency comb with a bandwidth 2.6 GHz, as shown in Figure 5b. The number of spectral lines in the ultra‐wide frequency comb is ten times that of the original frequency comb. Notably, the number of spectral lines in the ultra‐wide frequency comb can be further enhanced by increasing the number of probe tones.

The accuracy of the frequency comb is crucial for precision measurement and other applications. To enhance its accuracy, we have proposed a method to enhance its accuracy by subdividing the frequency comb. Specifically, the seed frequency comb is generated via an exact blue‐sideband pump at a fixed power of –3 dBm, ensuring the spectral stability of the comb is maintained. The fundamental subdivision unit of the frequency comb is defined by configuring the probe tone to operate at 1 dBm power and 6.52 GHz frequency, as shown in Figure 5c. The frequency interval between the probe tones is precisely given by $\omega_{m}$/15. Each probe tone and the seed frequency comb will individually generate an IFC through the third‐order cascaded nonlinear effect. As shown in Figure 5d, these IFCs are combined to form a frequency comb with an ultrafine comb tooth spacing, which is determined by the spacing of the probe tones. The accuracy of the cascaded frequency comb can be flexibly adjusted by altering the frequency interval between the probe tones. The proposed approach not only enhances the spectral precision of the frequency comb but also provides exceptional tunability. Through precise control of both the number of probe tones and their frequency interval, we can fine‐tune the spacing of the frequency comb. This unprecedented level of spectral engineering enables customized comb tooth spacing patterns for specialized applications requiring exact frequency‐match configurations.

## 3 Discussion

In this work, we have implemented an on‐demand and tunable IFC in a cascaded nonlinear system, which consists of a cavity electromechanical system and a HEMT working in the nonlinear regime. We have investigated the OMFC mediated by the mechanical mode in a cavity electromechanical device. This OMFC will serve as the seed signal for the HEMT and is mixed with another weak single‐frequency coherent tone to generate a new frequency comb. Different from the traditional micro‐cavity Kerr comb, the central frequency of the cascaded frequency comb is not limited by the device. Through cascaded third‐order nonlinear interactions, the generated IFC exhibits deterministic central frequency locking to the probe frequency. By increasing the number of probe tones and engineering their frequency intervals to satisfy certain conditions, we experimentally implemented a customized IFC through cascaded nonlinear effects. This approach enables deterministic customization over three key parameters: the central frequency of the comb, the comb teeth spacing (accuracy of the frequency comb), and the number of spectral lines.

The accurate control of the key parameters such as the central frequency, comb tooth spacing, and spectral line number can further improve the applicability and performance of the frequency combs in various applications. Our experiment shows that the cascaded nonlinear system is an ideal platform for achieving accurate control of the IFC parameters. An IFC, with the real‐time engineered central frequency, comb tooth spacing, and spectral line number, can be generated within this cascaded nonlinear system. As a prospect, increasing the number of spectral lines can enhance the applications of the frequency combs in SNR enhancement and frequency multiplexing [91, 92]. The ability to precisely control the comb tooth spacing and central frequency can enhance the performance and expand the application scope of the frequency combs in various fields such as atomic clock [32, 33, 34, 35, 36], acoustic detection [93, 94], LiDAR [95, 96, 97], and molecular fingerprint [40, 41, 98].

## Author Contributions

C. W. carried out the device design and fabrication. W. W. developed the device release recipe. Y. W and S. W. conducted the measurements. Y. L. conceived the project and coordinated the research. All authors analyzed the measurement results, drafted the manuscript, and gave their final approval for publication.

## Conflicts of Interest

The authors declare no conflicts of interest.

## Supporting information


**Supporting File 1**: advs77903‐sup‐0001‐SuppMat.pdf.

**Supporting File 2**: advs77903‐sup‐0002‐MovieS1.mp4.

## Data Availability

The datasets used and analyzed in this paper are available from the corresponding authors upon reasonable request.
