# Supplementary material for: Customized Itinerant Frequency Combs by Cascaded Nonlinear Effects
Source: Adv Sci (Weinh). 2026 Sep 27:e77903. Online ahead of print. doi: 10.1002/advs.77903 (PMC13616386; doi:10.1002/advs.77903)
Supplement: Supplementary file 1 [file ADVS-9999-e77903-s001.pdf]

Supporting Information

Customized itinerant Frequency Combs by Cascaded Nonlinear Effects

Yi Wang, Yulong Liu*, Zeji Li, Wenyan Wang, Sihan Wang, Cheng Wang, Jingwei Zhou, Jie-Qiao Liao, and Tiefu Li

Corresponding author: liuyl@baqis.ac.cn

In the following, we provide supporting documentation for our main manuscript. We first report on our experimental setup and then give the methodology to derive the security analysis.

S1. Fabrication of the device

As stated in the main text, our sample is a superconducting cavity electromechanical device (see the Figure S2b), which is composed of a superconducting microwave circuit resonator and a drum-type mechanical resonator (see the Figure S1a). Its design is similar to that reported in Refs. [S1–S3]. The mechanical oscillator is a circular aluminum drum of diameter 20 µm. Based on the stress and geometric structure of the aluminum drum mechanical resonator, its fundamental oscillation frequency is calculated to be 9.1 MHz. The embedded subfigure in Figure S3c shows the simulated mechanical oscillation field distribution of this structure.

Device fabrication begins on a quartz substrate using a multi-step electron-beam lithography (EBL) and deposition process (see the Figure S1b). First, standard EBL with a PMMA copolymer stack resist is performed, followed by electron-beam evaporation of a 40 nm aluminum (Al) layer to pattern the inductors, external coupler, bottom electrodes of the drums, connecting pads, and ground planes. Next, a 75 nm amorphous silicon (α-Si) sacrificial layer is deposited via PECVD, patterned with ma-N 2403 negative-tone resist, and etched using SF_6 plasma RIE to position it directly above the bottom electrodes. Finally, a concluding lithography step defines the 120 nm-thick top Al drumheads, which overlap the sacrificial layer and connect to the ground lines, preparing the structure for subsequent sacrificial layer release.

After the substrate was diced into 4 $\times$ 4 mm chips, the sacrificial layer was removed via isotropic SF_6 plasma reactive-ion etching (RIE), yielding an approximately 80 nm vacuum gap. The electromechanical device was then mounted at the geometric center of an aluminum sample box using a low-temperature adhesive (EL11). Finally, an antenna was employed to read out the sample data.

S2. Experimental setup

Detailed cryogenic setup, as well as room temperature microwave devices is shown in Figure S2a. The arrows indicate the direction of the microwave signal. In the input line, attenuators equipped according to different temperature zones are used to reduce background noise. The circulator is positioned between the sampler and the input line, while an isolator and a directional coupler are incorporated into the output line. These configurations help collectively avoid back-reflections of microwave signals. The Vector Network analyzer (VNA) injects a probe signal (marked by the blue arrow) into the channel to obtain the basic characteristic parameters of the electromechanical device. Figure S3a shows the S parameters of the microwave cavity, which are obtained by inputting the -30 dBm probe signal using the VNA. From these parameters, the resonant frequency $\omega_c/2\pi = 4.959486$ GHz and damping rate $\kappa/2\pi = 346.2$ kHz of the microwave resonator can be derived. This suggests that the quality factor of this microwave cavity reaches 1.43×10^4. The Spectrum Analyzer (RSA) serves as the core instrument for spectral characterization, delivering comprehensive frequency-domain analysis and time-correlated measurements of the device under test (DUT). The Precision Signal Generator (PSG) functions as the pump excitation source, where parametric control of amplitude and frequency enables precise modulation of the cavity fields. This cavity field couples to the suspended drum membrane, which in turn exerts back-action on the cavity resonant frequency through displacement-dependent frequency shifts and thus accounts for cavity photon number fluctuation. This mechanism induces Kerr-like nonlinearity.

The basic characteristics of the drum-type mechanical resonator are measured through optomechanically induced transparency (OMIT), where coherent optomechanical interaction between the microwave cavity field and aluminum drum mechanical resonator manifests as the narrow absorption (OMIA) or transparency (OMIT) window within the microwave cavity resonant spectrum. The intrinsic frequency of the mechanical mode can be obtained based on the OMIT phenomenon. This process involves the strong pump signal near the frequency $\omega_d = \omega_c - \omega_m$ and the weak probe signal near the cavity resonant frequency ω_c. Under the resolved sideband condition ($\omega_m > \kappa$), the radiation pressure from the pump and probe signals drives coherent motion of the mechanical resonator. This coherent motion mediates the conversion between the optical sidebands, enabling precise detection of the mechanical

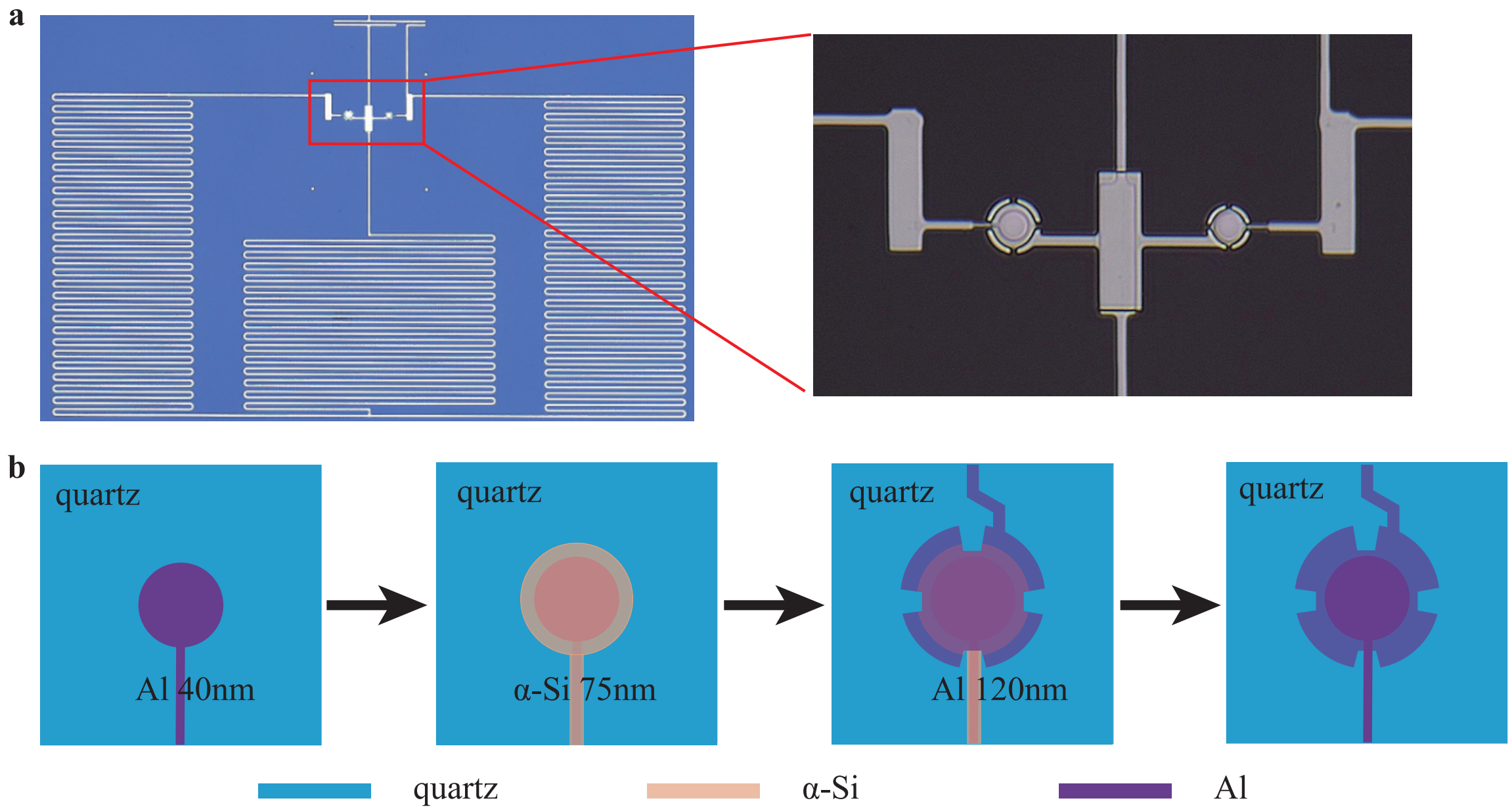

Figure S1. **a** The micrograph of the microwave optomechanical device shows the entire chip and a magnified view of the drum-like structure. **b** Process flow for fabricating the superconducting electromechanical devices.

resonator through the modulation of the probe signal. As shown in Figure S3b, the detailed spectrum of the cavity transparency generation at the pump power of 12 dBm is presented. The fundamental oscillation frequency of the mechanical mode is 9.148 MHz. As shown in Figure S3c, the power spectral density of the mechanical mode can be obtained by RSA via IQ mixing based frequency down-conversion. The linewidth of the resonant peak is 103.15 Hz after Lorentzian fitting. This suggests that the quality factor of this mechanical resonator reaches 8.86×10^4.

S3. Single photon coupling strength

In cavity electromechanical systems, the optomechanical coupling strength between the microwave resonator mode and the mechanical mode scales with the square root of the cavity photon number. However, the actual focus is the optomechanical coupling strength between a single photon and a single phonon, which is known as the single-photon coupling strength g. The value of g determines the performance of the electromechanical system. The single-photon coupling strength can be precisely quantified by comparing the spectrum of the thermomechanical sideband with the spectrum of a reliable calibration tone at a calibrated temperature, where the thermomechanical sideband is thermally determined.

The dilution refrigerator provides an ultra-low temperature environment essential for electromechanical devices, yet the effective temperature of the mechanical mode cannot be reliably determined through conventional measurements due to the presence of thermal excitations. To address this, we introduce a controlled heat source to modify the refrigerator's thermal equilibrium and observe the spectral changes of the mechanical mode. This technique enables us to establish a calibration between the observed spectrum and the corresponding phonon occupation number of the mechanical mode. As shown in Figure S4a, the spectral area exhibits a linear relationship with temperature above $T \approx 26$ mK, while the dilution fridge temperature is typically around 10 mK without a heating source. Therefore, it is reasonable for us to choose an ambient temperature of 31 mK to test the single-photon coupling strength g.

Based on the spectral characteristics of the mechanical mode, we experimentally derived the single-photon coupling strength g when the calibration tone and the pump signal were injected. The calibration signal is set at a position with a slight detuning from the blue sideband $\omega_c + \omega_m$ and outside the mechanical linewidth. Consequently, the RSA would observe two output signals: the thermomechanical sideband [see Figure S4b] generated by the mechanical mode and the calibration tone [see Figure S4c]. Using the calibration tone as a reference, the single-photon coupling strength in the electromechanical system can be expressed as

$$g = \frac{r}{2n_m}\left(\frac{\kappa}{\kappa_e}\right)^2\left(\omega_m^2 + \frac{\kappa^2}{2}\right)\frac{P_{\text{SB}}}{P_{\text{cali}}}, \tag{S1}$$

where r represents the ratio of input power between the calibration tone and the pump tone. $n_m = K_B T/(\hbar\omega_m)$ denotes the phonon number under $T = 31$ mK, and K_B is the Boltzmann constant. P_{SB} and P_{cali} indicate the detected

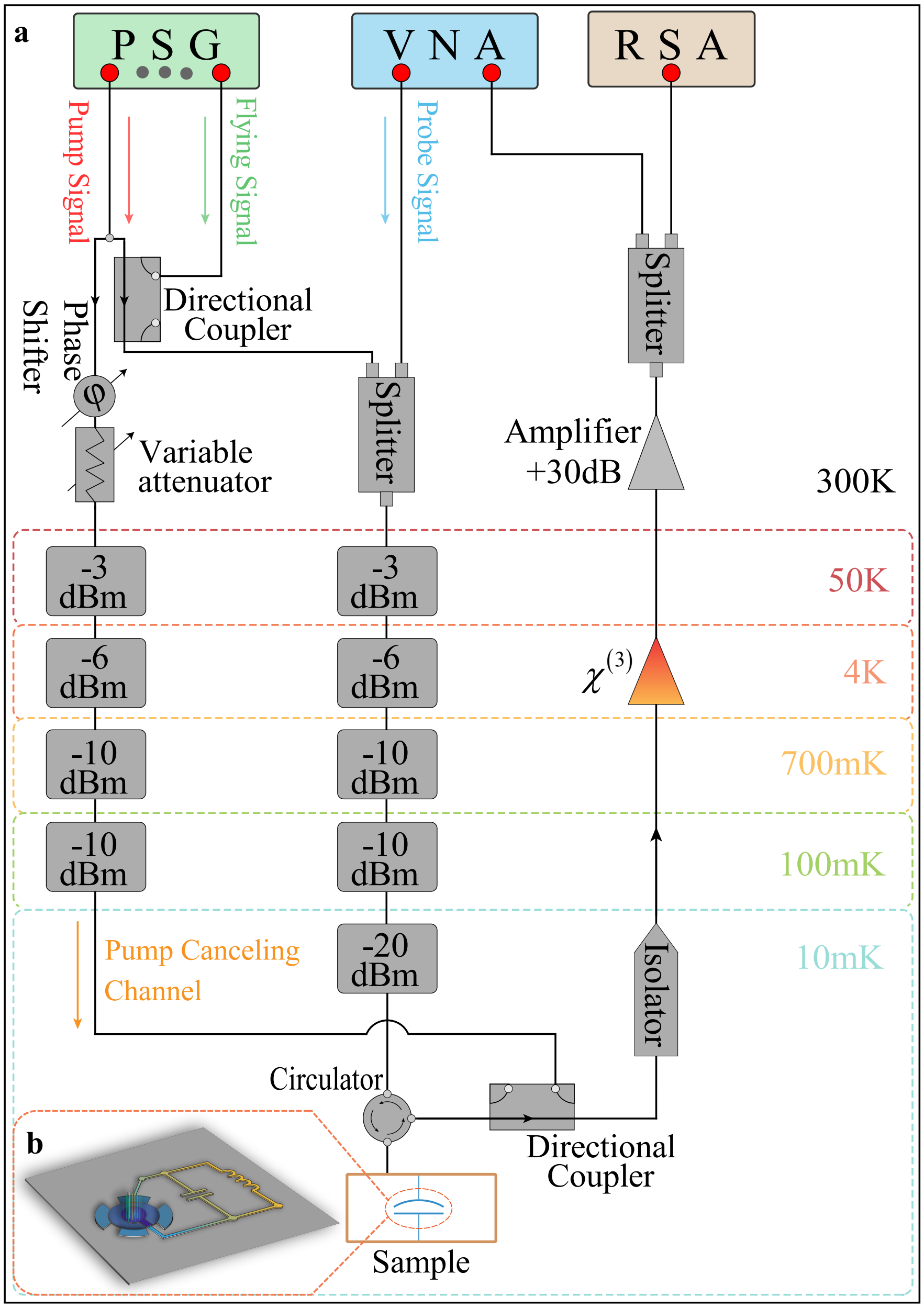

Figure S2. **a** Experimental setup. Sideband pumps are injected to study nonlinear cavity dynamics. **b** The circuit model diagram of the electromechanical system. The blue structure represents the aluminum drum mechanical resonator.

powers of the thermomechanical sideband and the calibration tone, which are measured by RSA. The ratio of these two powers is equal to the ratio of the areas under the mechanical peak and the modulation peak. Consequently, we can obtain the single-photon coupling strength $g/2\pi \approx 62.49$ Hz.

The basic performance parameters of the microwave optomechanical device were obtained by the above method, as listed in Table S1.

S4. Generation of optomechanical frequency combs

As mentioned in the main text, we consider a cavity electromechanical system consisting of superconducting circuits (microwave resonators in the radio frequency range) coupled to an aluminum drum-type mechanical resonator. The microwave cavity field is strongly driven by the pump tone with frequency ω_d and amplitude S_{in}. The total Hamiltonian of the system can be expressed as

$$H_{sys}/\hbar = \omega_c a^\dagger a + \omega_m b^\dagger b - g a^\dagger a \left(b^\dagger + b\right) + i\sqrt{\kappa_e} S_{in} \left(a^\dagger e^{-i\omega_d t} - a e^{i\omega_d t}\right), \tag{S2}$$

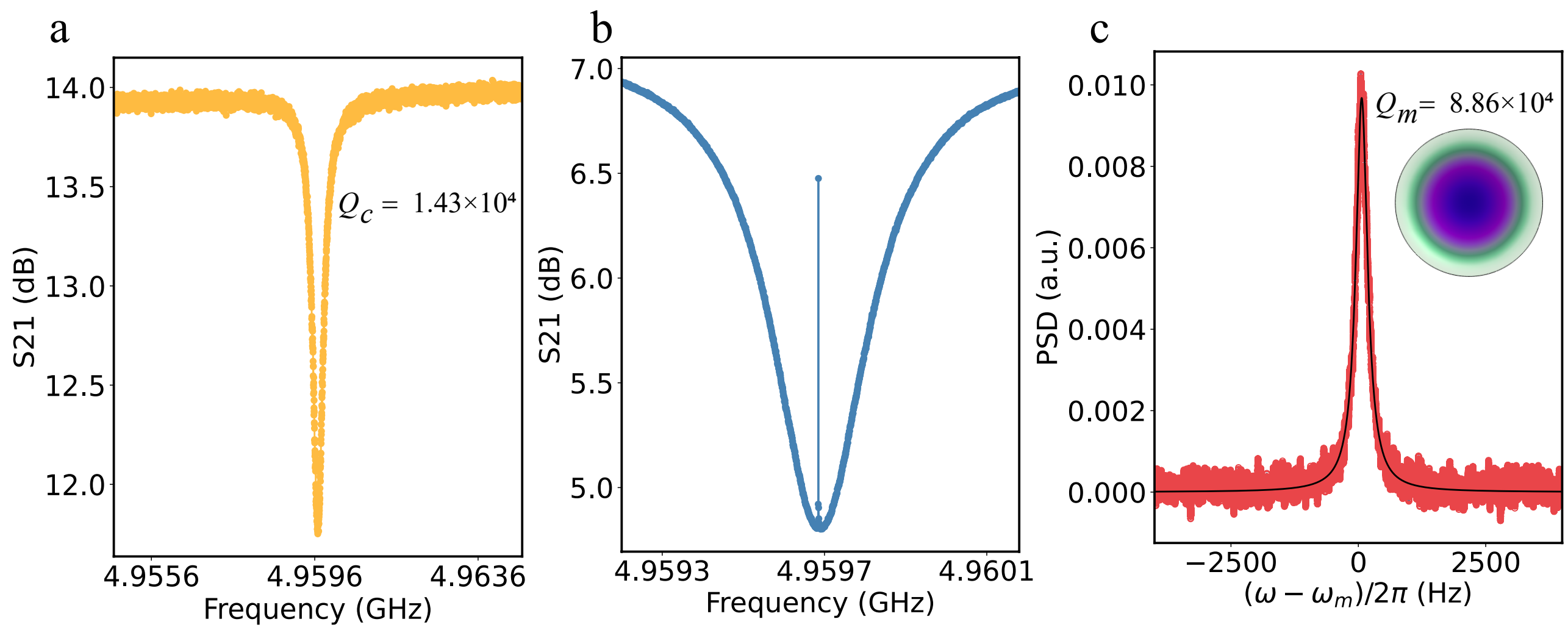

Figure S3. **a** Measurement of the S21 parameter of the microwave cavity mode. **b** The mechanical mode induced cavity transparency. **c** Measured power spectral density of the mechanical mode.

Table S1. Device parameters

**Parameter**	**Symbol**	**Value**
Cavity frequency	ω_c	$2\pi \times 4.959486$ GHz
Cavity external coupling rate	$\kappa_{\rm e}$	$2\pi \times 38.3$ kHz
Cavity internal loss rate	$\kappa_{\rm i}$	$2\pi \times 307.9$ kHz
Cavity damping rate	$\kappa = \kappa_{\rm e} + \kappa_{\rm i}$	$2\pi \times 346.2$ kHz
Cavity quality factor	$Q_{\rm c}$	1.43×10^4
Mechanical frequency	ω_m	$2\pi \times 9.148$ MHz
Mechanical damping rate	γ_m	$2\pi \times 103.15$ Hz
Mechanical quality factor	Q_m	8.86×10^4
Single-photon coupling rate	g	$2\pi \times 62.49$ Hz

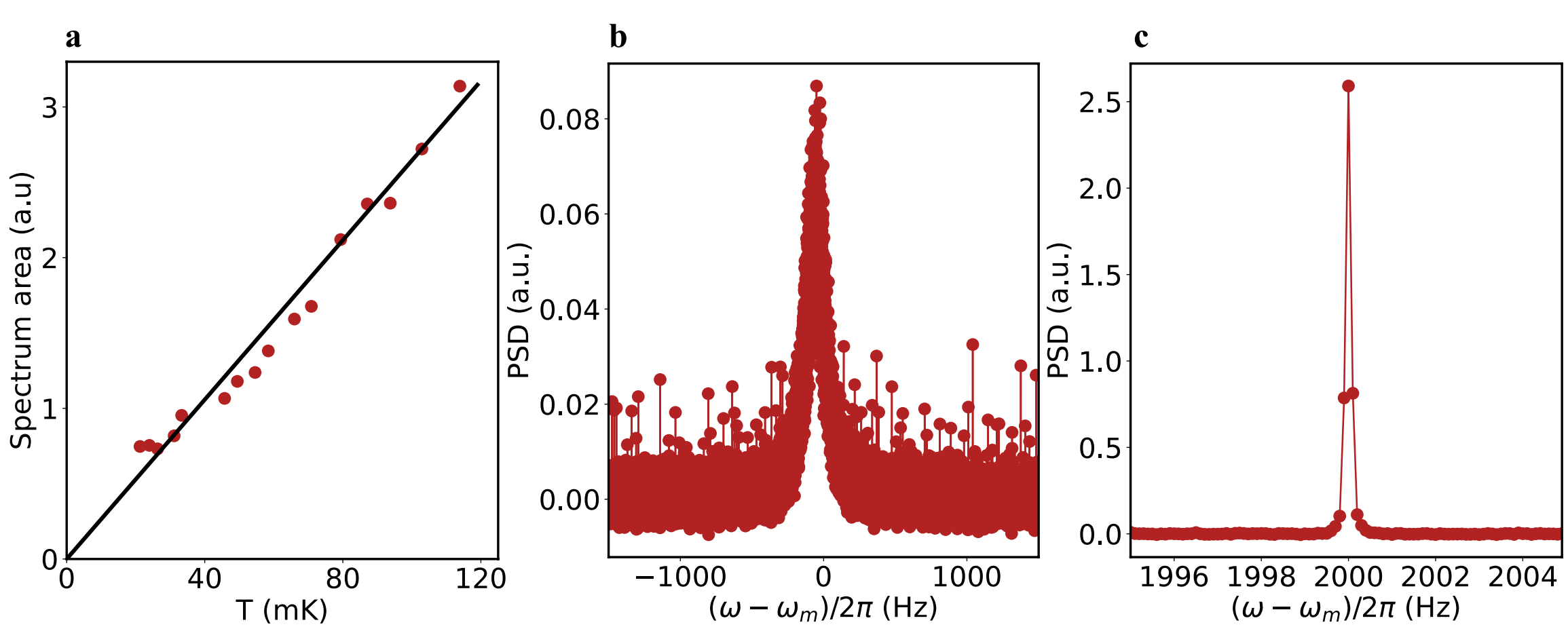

Figure S4. **a** Temperature dependency of spectrum area for the mechanical mode (red data). Black lines are linear fitting. **b** Detected spectrum of the mechanical mode mediated thermomechanical sideband. **c** Detected spectrum of the mechanical mode targeted calibration tone.

where a ($a^\dagger$) and b ($b^\dagger$) are the annihilation (creation) operators of the microwave mode and the mechanical mode with resonance frequencies ω_c and ω_m, respectively. The parameter g represents the single-photon coupling strength between the mechanical mode and the microwave mode. The coefficient κ is the cavity damping rate, which includes the coupling rate κ_e to the external field and the intrinsic loss κ_i, so that $\kappa = \kappa_i + \kappa_e$.

According to the Supplementary Equation (S2), the equation of motion for the electromechanical system can be written

$$\frac{da}{dt} = -i\left(\omega_c - g\left(b^\dagger + b\right) - \frac{\kappa}{2}\right)a + \sqrt{\kappa_e}S_{in}e^{-i\omega_d t}, \tag{S3}$$

$$\frac{db}{dt} = -\left(i\omega_m + \frac{\gamma}{2}\right)b + iga^\dagger a. \tag{S4}$$

where γ is the decay rate of the mechanical mode.

When the power of the pump tone exceeds a certain threshold, the system will enter into coupled oscillations between the microwave resonance mode and the mechanical mode. The general oscillation form of the mechanical oscillator is $b(t) = B_0 \exp\left[-i\left(\omega_m t + \pi/2\right)\right]$, where B_0 denotes the oscillation amplitude. By substituting the trial solution of the mechanical oscillator into the Supplementary Equation (S3), the dynamical evolution of the microwave field can be given by

$$\frac{da}{dt} = -\left(i\omega_c - ig\left[B_0\exp\left(i\omega_m t - \pi/2\right) + B_0\exp\left(-i\omega_m t - \pi/2\right)\right] + \frac{\kappa}{2}\right)a + \sqrt{\kappa_e}S_{in}e^{-i\omega_d t} \tag{S5}$$

Utilizing the series expansion of the Bessel function, i.e., $\exp(i\xi\cos\phi) = \sum_{k=-\infty}^{\infty} i^k J_k(\xi)\exp(ik\phi)$, the dynamical equation of the microwave field can be obtained

$$\begin{aligned} a(t) &= \sqrt{\kappa_e}S_{in}\sum_{l=-\infty}^{\infty} i^l J_l(\xi)\, e^{il\omega_m t}e^{-\frac{\kappa}{2}t - i\omega_c t}\int e^{-i\omega_d t}\sum_{k=-\infty}^{\infty} i^k J_k(-\xi)\, e^{ik\omega_m t}e^{\frac{\kappa}{2}t + i\omega_c t}dt \\ &= \sqrt{\kappa_e}S_{in}\sum_{l=-\infty}^{\infty} i^l J_l(\xi)\, e^{il\omega_m t}e^{-\frac{\kappa}{2}t - i\omega_c t}\sum_{k=-\infty}^{\infty} i^k J_k(-\xi)\int e^{-i\omega_d t}e^{ik\omega_m t}e^{\frac{\kappa}{2}t + i\omega_c t}dt \\ &= \sqrt{\kappa_e}S_{in}\sum_{l=-\infty}^{\infty} i^l J_l(\xi)\, e^{il\omega_m t}\frac{\sum_{k=-\infty}^{\infty} i^k J_k(-\xi)}{\kappa/2 - i\Delta_{dc} + ik\omega_m}e^{-i\omega_d t}e^{ik\omega_m t} \\ &= \sum_{n=-\infty}^{\infty}\sum_{k=-\infty}^{\infty}\sqrt{\kappa_e}S_{in}i^n\frac{J_{n-k}(\xi)\, i^k J_k(-\xi)}{\kappa/2 - i\Delta_{dc} + ik\omega_m}e^{in\omega_m t}e^{-i\omega_d t}, \end{aligned}$$

where the detuning is defined as $\Delta_{dc} = \omega_d - \omega_c$. $J_k(\xi)$ is the Bessel function of the first kind and $\xi = 2gB_0/\omega_m$. Therefore, we can simplify the cavity field solution as

$$a(t) = \sum_{n=-\infty}^{\infty} a_n e^{-i\omega_d t}e^{in\omega_m t}, \tag{S6}$$

where

$$a_n = \sqrt{\kappa_e}S_{in}\sum_{k=-\infty}^{\infty} i^n\frac{J_{n-k}(\xi)\, J_k(-\xi)}{ik\omega_m - i\Delta_{dc} + \kappa/2}. \tag{S7}$$

This equation well describes the characteristics of the optomechanical combs mediated by the mechanical oscillator. Using the input-output relationship $a_{out} = a_{in} - \sqrt{\kappa_e}a$, the output field of the electromechanical system is given by

$$\begin{aligned} a_{out}(t) &= a_{in}(t) - \sqrt{\kappa_e}a(t) \\ &= S_{in}e^{-i\omega_d t} - \sqrt{\kappa_e}\sum_{n=-\infty}^{\infty} a_n e^{-i\omega_d t}e^{in\omega_m t} \\ &= S_{in}e^{-i\omega_d t} - \sqrt{\kappa_e}a_0 e^{-i\omega_d t} - \sqrt{\kappa_e}\sum_{n\neq 0} a_n e^{-i\omega_d t}e^{in\omega_m t} \\ &= \sum_{n=-\infty}^{\infty} a_{out,n}e^{-i\omega_d t}e^{in\omega_m t}, \end{aligned} \tag{S8}$$

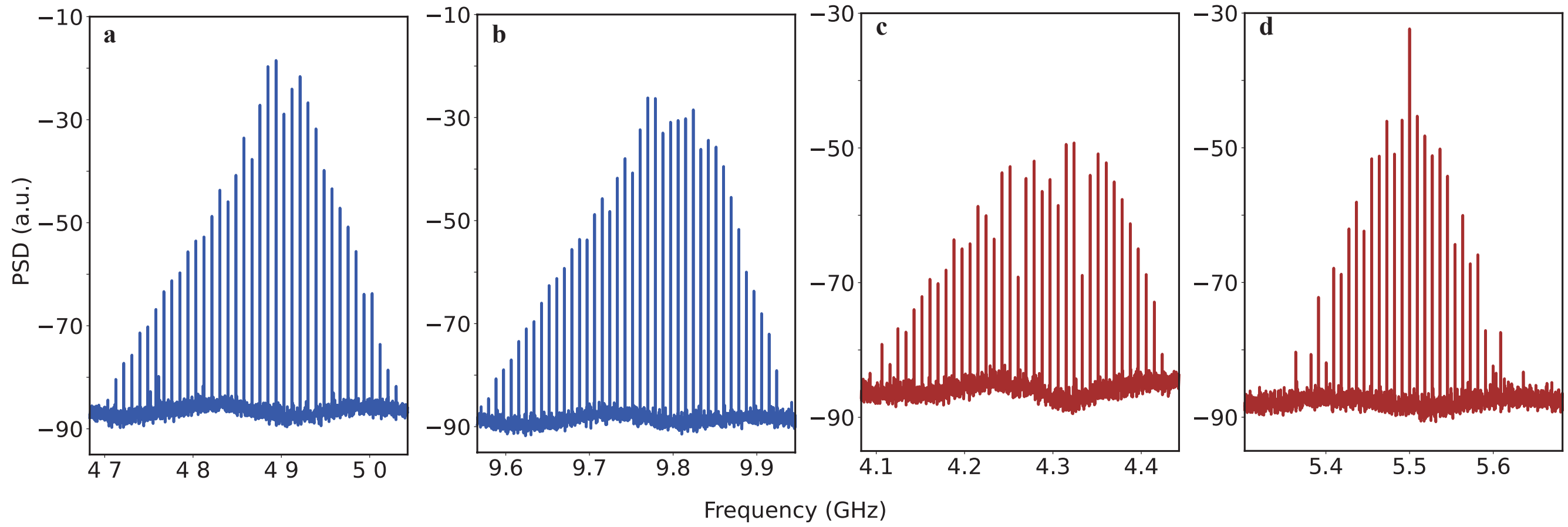

Figure S5. **a** Initial optomechanical frequency comb. **b** The OMFC generated at double frequency position after mixing by second order nonlinear polarization effect. **c** The IFC with central frequency $2\omega_d - \omega_p$. **d** The IFC with central frequency ω_p.

where

$$a_{out,0} = S_{in} - \sqrt{\kappa_e} a_0, \tag{S9a}$$

$$a_{out,n} = \sqrt{\kappa_e} a_n \quad (n \neq 0)\,, \tag{S9b}$$

According to the cavity field solution $a(t)$ [the Supplementary Equation (S6)], the expression of the electric field is given by

$$E_{out} = \sum_{n=-\infty}^{\infty} E_n a_{out,n} e^{-i(\omega_d - n\omega_m)t} + c.c.. \tag{S10}$$

Here, $E_n = \sqrt{\hbar(\omega_d - n\omega_m)/(V\varepsilon_0)}$ with V and ε_0 being the volume of the microwave field and vacuum permittivity, respectively.

S5. Generation of itinerant frequency combs

In this experiment, the cascaded nonlinear system consists of a superconducting cavity electromechanical device and a high electron mobility transistor (HEMT) working in the nonlinear regime. The output of the electromechanical system (the OMFC with the central frequency ω_d and tooth spacing ω_m) serves as the seed combs injected into the second nonlinear device. Under the second order nonlinear polarization effect, the second order polarization intensity $P^{(2)}(t)$ related to the seed signal E_{out} can be written as

$$\begin{aligned} P^{(2)}(t) &= \varepsilon_0 \chi^{(2)} E_{out} E_{out} \\ &= \sum_{n,l=-\infty}^{\infty} \varepsilon_0 \chi^{(2)} (\omega_d - n\omega_m, \omega_d - l\omega_m) E_n a_{out,n} E_l a_{out,l} e^{-i[2\omega_d - (n+l)\omega_m]t} \\ &\quad + \sum_{n,l=-\infty}^{\infty} \varepsilon_0 \chi^{(2)} (-\omega_d + n\omega_m, -\omega_d + l\omega_m) E_n^* a_{out,n}^* E_l^* a_{out,l}^* e^{i[2\omega_d - (n+l)\omega_m]t} \\ &\quad + \sum_{n,l=-\infty}^{\infty} \varepsilon_0 \chi^{(2)} (-\omega_d + n\omega_m, \omega_d - l\omega_m) E_n^* a_{out,n}^* E_l a_{out,l} e^{-i(n-l)\omega_m t} \\ &\quad + \sum_{n,l=-\infty}^{\infty} \varepsilon_0 \chi^{(2)} (\omega_d - n\omega_m, -\omega_d + l\omega_m) E_n a_{out,n} E_l^* a_{out,l}^* e^{-i(l-n)\omega_m t}. \end{aligned} \tag{S11}$$

where $\chi^{(2)}$ is the second-order polarizability. Using the reality condition of the second-order polarizability

$$\left[\chi^{(2)} (\omega_a, \omega_b)\right]^* = \chi^{(2)} (-\omega_a, -\omega_b)\,. \tag{S12}$$

The Equation (S11) can be simplified to

$$P^{(2)}(t) = \sum_{n,l} \varepsilon_0 \left(\chi_1^{(2)} E_n a_{out,n} E_l a_{out,l} e^{-i[2\omega_d - (n+l)\omega_m]t} + \chi_2^{(2)} E_n a_{out,n} E_l^* a_{out,l}^* e^{-i(l-n)\omega_m t} + c.c \right) \tag{S13}$$

where

$$\chi_1^{(2)} = \chi^{(2)}(\omega_d - n\omega_m, \omega_d - l\omega_m), \tag{S14a}$$

$$\chi_2^{(2)} = \chi^{(2)}(-\omega_d + n\omega_m, \omega_d - l\omega_m). \tag{S14b}$$

The OMFC [see Figure S5a] can produce new frequency components through mixing via the cascaded nonlinear effect, forming a new frequency comb. The first term indicates a series of beat frequencies with a spacing ω_m. The second term represents the generation of a new frequency comb with a central frequency $2\omega_d$ and a comb tooth spacing ω_m, which is called the frequency-doubled optomechanical comb, as shown in Figure S5b.

To explore the effect of cascading nonlinearity on the OMFC, the seed comb is injected into the second nonlinear device along with a weak single-frequency coherent tone $E_{fly} = E_p \exp(-i\omega_p t) + c.c.$. Through the third-order nonlinear polarization effect, the third-order nonlinear polarization intensity $P^{(3)}(t)$ related to the seed signal E_{out} and coherent tone E_{fly} is given by

$$\begin{aligned}
P^{(3)}(t) &= \varepsilon_0 \chi^{(3)} E_{out} E_{out} E_{fly} \\
&= 3\varepsilon_0 \sum_{n,l} \chi^{(3)}(\omega_d - n\omega_m, \omega_d - l\omega_m, \omega_p) E_n a_{out,n} E_l a_{out,l} E_p e^{-i(2\omega_d + \omega_p - (n+l)\omega_m)t} \\
&\quad + 3\varepsilon_0 \sum_{n,l} \chi^{(3)}(-\omega_d + n\omega_m, -\omega_d + l\omega_m, -\omega_p) E_n^* a_{out,n}^* E_l^* a_{out,l}^* E_p^* e^{i(2\omega_d + \omega_p - (n+l)\omega_m)t} \\
&\quad + 3\varepsilon_0 \sum_{n,l} \chi^{(3)}(-\omega_d + n\omega_m, \omega_d - l\omega_m, \omega_p) E_n^* a_{out,n}^* E_l a_{out,l} E_p e^{-i(\omega_p + (n-l)\omega_m)t} \\
&\quad + 3\varepsilon_0 \sum_{n,l} \chi^{(3)}(\omega_d - n\omega_m, -\omega_d + l\omega_m, -\omega_p) E_n a_{out,n} E_l^* a_{out,l}^* E_p^* e^{i(\omega_p + (n-l)\omega_m)t} \\
&\quad + 3\varepsilon_0 \sum_{n,l} \chi^{(3)}(\omega_d - n\omega_m, -\omega_d + l\omega_m, \omega_p) E_n a_{out,n} E_l^* a_{out,l}^* E_p e^{-i(\omega_p - (n-l)\omega_m)t} \\
&\quad + 3\varepsilon_0 \sum_{n,l} \chi^{(3)}(-\omega_d + n\omega_m, \omega_d - l\omega_m, -\omega_p) E_n^* a_{out,n}^* E_l a_{out,l} E_p^* e^{i(\omega_p - (n-l)\omega_m)t} \\
&\quad + 3\varepsilon_0 \sum_{n,l} \chi^{(3)}(\omega_d - n\omega_m, \omega_d - l\omega_m, -\omega_p) E_n a_{out,n} E_l a_{out,l} E_p^* e^{-i(2\omega_d - \omega_p - (n+l)\omega_m)t} \\
&\quad + 3\varepsilon_0 \sum_{n,l} \chi^{(3)}(-\omega_d + n\omega_m, -\omega_d + l\omega_m, \omega_p) E_n^* a_{out,n}^* E_l^* a_{out,l}^* E_p e^{i(2\omega_d - \omega_p - (n+l)\omega_m)t},
\end{aligned} \tag{S15}$$

where the parameters 3 is the result of considering the intrinsic commutation symmetry of the third-order polarizability and $\chi^{(3)}$ is third-order polarizability. Considering the intrinsic commutation symmetry of the third-order polarizability

$$\left[\chi^{(3)}(\omega_a, \omega_b, \omega_c)\right]^* = \chi^{(3)}(-\omega_a, -\omega_b, -\omega_c). \tag{S16}$$

We can simplify the third-order nonlinear polarization intensity as

$$\begin{aligned}
P^{(3)}(t) &= 3\varepsilon_0 \sum_{n,l} \chi^{(3)}(-\omega_d + n\omega_m, \omega_d - l\omega_m, \omega_p) E_n^* a_{out,n}^* E_l a_{out,l} E_p e^{-i(\omega_p + (n-l)\omega_m)t} \\
&\quad + 3\varepsilon_0 \sum_{n,l} \chi^{(3)}(\omega_d - n\omega_m, -\omega_d + l\omega_m, \omega_p) E_n a_{out,n} E_l^* a_{out,l}^* E_p e^{-i(\omega_p - (n-l)\omega_m)t} \\
&\quad + 3\varepsilon_0 \sum_{n,l} \chi^{(3)}(\omega_d - n\omega_m, \omega_d - l\omega_m, -\omega_p) E_n a_{out,n} E_l a_{out,l} E_p^* e^{-i(2\omega_d - \omega_p - (n+l)\omega_m)t} \\
&\quad + 3\varepsilon_0 \sum_{n,l} \chi^{(3)}(\omega_d - n\omega_m, \omega_d - l\omega_m, \omega_p) E_n a_{out,n} E_l a_{out,l} E_p e^{-i(2\omega_d + \omega_p - (n+l)\omega_m)t} + c.c.,
\end{aligned} \tag{S17}$$

It can be found that the OMFC and the probe tone generate new frequency components through third-order nonlinear polarization effects. The first and second terms correspond to the generation of a new frequency comb with the central

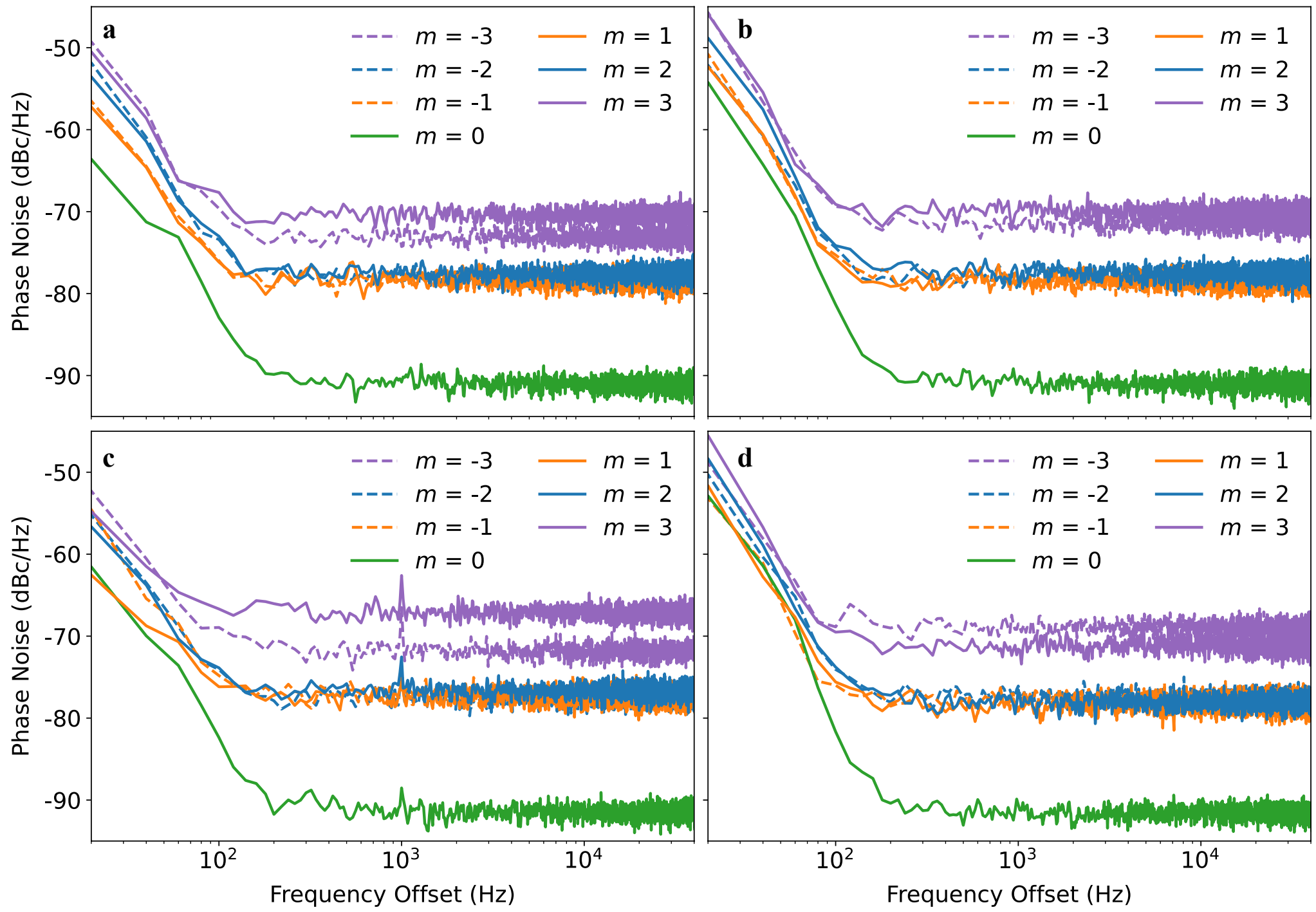

Figure S6. Phase noise spectra of the IFC. Phase noise profiles measured under four distinct input configurations with varying phase noise levels of the pump and probe tones: **a** low-phase-noise pump and low-phase-noise probe; **b** low-phase-noise pump and high-phase-noise probe; **c** high-phase-noise pump and low-phase-noise probe; and **d** high-phase-noise pump and high-phase-noise probe. The other parameters include a pump tone power of 20 dBm, a probe tone power of −20 dBm, and a center frequency of 7 GHz for the IFC.

frequency ω_p and the tooth spacing ω_m, which is called the IFC, as shown in Figure S5d. The third term indicates the frequency comb of the central frequency $2\omega_d - \omega_p$ and the comb tooth spacing ω_m, as shown in Figure S5c. The fourth term generates the frequency comb with higher frequencies $2\omega_d + \omega_p$, exceeding the measurable bandwidth of 4-8 GHz, and therefore is not discussed.

In this experiment, we focus on the mechanism responsible for generating the new frequency comb with the central frequency ω_p and the tooth spacing ω_m. The third-order nonlinear polarization intensity $P_f^{(3)}(t)$ can be expressed as

$$P_f^{(3)}(t) = 3\varepsilon_0 \sum_{n,l} \{\Gamma_1^{(3)} E_n^* E_l E_p e^{-i[\omega_p + (n-l)\omega_m]t} + \Gamma_2^{(3)} E_n E_l^* E_p e^{-i[\omega_p - (n-l)\omega_m]t} + c.c.\} \tag{S18}$$

where

$$\Gamma_1^{(3)} = \chi^{(3)}(-\omega_d + n\omega_m, \omega_d - l\omega_m, \omega_p) a_{out,n}^* a_{out,l}, \tag{S19a}$$

$$\Gamma_2^{(3)} = \chi^{(3)}(\omega_d - n\omega_m, -\omega_d + l\omega_m, \omega_p) a_{out,n} a_{out,l}^* \tag{S19b}$$

This Equation $P_f^{(3)}(t)$ explains the physical mechanism that the optomechanical frequency and the probe tone generate the IFCs through the third-order nonlinear polarization effect.

S6. Phase noise of itinerant frequency combs

Owing to inadvertent damage sustained by the original sample during thermal cycling in the dilution refrigerator, a replacement sample was employed for subsequent phase-noise characterization. All measurement protocols were kept strictly identical to those used for the original sample. The new sample features a microwave mode with a linewidth of 85 kHz centered at 5.409 GHz, and a mechanical oscillator with a linewidth of 3 Hz at 1.9 MHz. The single-photon coupling strength is determined to be 23 Hz.

We estimate the single-sideband (SSB) phase-noise power spectral density using Welch's method applied to the unwrapped phase fluctuations extracted from the demodulated in-phase and quadrature components. Since the

microwave source used in our experiment does not allow direct adjustment of its phase noise, we employed two signal generators of different models: a low-phase-noise source (SLFS0208T, nominal phase noise of −130 dBc/Hz @ 10 kHz) and a high-phase-noise source (SLFS0218F, nominal phase noise of −115 dBc/Hz @ 10 kHz), to provide two distinct phase noise floors.

Figure S6 compares the phase noise spectra of the IFC across four distinct pump–probe noise configurations. First, comparing cases with identical probe tones but differing pump noise levels (Figure S6a and c) reveals virtually indistinguishable noise spectra. This insensitivity confirms that pump phase noise has a negligible impact, as the comb's phase fluctuations are predominantly dictated by modulation noise associated with fluctuations of the intrinsic mechanical resonance frequency. In contrast, comparing low-phase-noise probe cases (a, c) directly with high-phase-noise probe cases (b, d) demonstrates a severe 5–8 dBc/Hz degradation in close-in phase noise. This indicates that low-frequency phase fluctuations of the probe—induced by phase walk-off—are directly mapped onto the comb via cascaded $\chi^{(3)}$ nonlinear mixing, making probe coherence the primary limit on the comb's intrinsic linewidth. Second, across all four subfigures, the sideband spectra consistently feature near-perfect overlap between symmetrical positive and negative orders ($+m$ and $-m$, indicated by solid and dashed lines), alongside a stepwise elevation with increasing $|m|$ relative to the central carrier ($m = 0$, green curve). This universal behavior confirms that, regardless of the pump/probe noise levels, higher-order sidebands coherently accumulate and linearly map the phase jitter of the intrinsic mechanical resonance ($\delta\phi_m = m\delta\theta$).

References

[S1] L. M. Lépinay, C. F. Ockeloen-Korppi, M. J. Woolley, and M. A. Sillanpää, Quantum mechanics–free subsystem with mechanical oscillators. *Science* **2021**, *372*, 625.

[S2] C. Wang, L. Banniard, K. Børkje, F. Massel, L. M. de Lépinay, and M. A. Sillanpää, Ground-state cooling of a mechanical oscillator by a noisy environment. *Nat. Commun.* **2024**, *15*, 7395.

[S3] S. Wang, C. Wang, M. H. J. de Jong, L. Mercier de Lépinay, J. Zhou, M. A. Sillanpää, and Y. Liu, Pump-threshold-free frequency comb via cavity Floquet engineering. *Nat. Commun.* **2026**, *17*, 2453.
